# KNSTRN Is a Prognostic Biomarker That Is Correlated with Immune Infiltration in Breast Cancer and Promotes Cell Cycle and Proliferation

**DOI:** 10.1007/s10528-023-10615-2

**Published:** 2024-01-10

**Authors:** Wenwu Zhang, Yuhan Xiao, Quan Zhou, Xin Zhu, Yanxia Zhang, Qin Xiang, Shunhong Wu, Xiaoyu Song, Junxiu Zhao, Ruanfei Yuan, Bin Xiao, Linhai Li

**Affiliations:** 1 Department of Laboratory Medicine, The Sixth Affiliated Hospital of Guangzhou Medical University, Qingyuan People’s Hospital, Qingyuan, 511518 China; 2 Department of Laboratory Medicine, The Affiliated Suzhou Hospital of Nanjing Medical University, Suzhou Municipal Hospital, Gusu School, Nanjing Medical University, Suzhou, 215008 China; 3 School of Public Health, Dali University, Dali, 671000 China; 4 Department of Laboratory Medicine, General Hospital of Southern Theater Command of People’s Liberation Army (PLA), Guangzhou, 510010 China

**Keywords:** KNSTRN, Breast cancer, Prognosis biomarker, Immune infiltration, Cell cycle

## Abstract

Kinetochore-localized astrin/SPAG5-binding protein (KNSTRN) promotes the progression of bladder cancer and lung adenocarcinoma. However, its expression and biological function in breast cancer remain largely unknown. Therefore, this study aimed to analyze KNSTRN expression, prognoses, correlation with immune infiltration, expression-associated genes, and regulated signaling pathways to characterize its role in regulating the cell cycle using both bioinformatics and in vitro functional experiments. Analyses of The Cancer Genome Atlas, Gene Expression Omnibus, TIMER, and The Human Protein Atlas databases revealed a significant upregulation of KNSTRN transcript and protein levels in breast cancer. Kaplan–Meier survival analyses demonstrated a significant association between high expression of KNSTRN and poor overall survival, relapse-free survival, post-progression survival, and distant metastases-free survival in patients with breast cancer. Furthermore, multivariate Cox regression analyses confirmed that KNSTRN is an independent prognostic factor for breast cancer. Immune infiltration analysis indicated a positive correlation between KNSTRN expression and T regulatory cell infiltration while showing a negative correlation with Tgd and natural killer cell infiltration. Gene set enrichment analysis along with single-cell transcriptome data analysis suggested that KNSTRN promoted cell cycle progression by regulating the expression of key cell cycle proteins. The overexpression and silencing of KNSTRN in vitro, respectively, promoted and inhibited the proliferation of breast cancer cells. The overexpression of KNSTRN enhanced the expression of key cell cycle regulators, including CDK4, CDK6, and cyclin D3, thereby accelerating the G1/S phase transition and leading to aberrant proliferation of breast cancer cells. In conclusion, our study demonstrates that KNSTRN functions as an oncogene in breast cancer by regulating immune response, promoting G1/S transition, and facilitating breast cancer cell proliferation. Moreover, KNSTRN has potential as a molecular biomarker for diagnostic and prognostic prediction in breast cancer.

**Supplementary Information:**

The online version contains supplementary material available at 10.1007/s10528-023-10615-2.

## Introduction

According to the 2020 Global Cancer Burden data from the World Health Organization’s International Agency for Research on Cancer, breast cancers are not only the most common tumors in the world (Ferlay et al. 2021) but are also the leading cause of cancer-related deaths among women worldwide (Siegel et al. 2019). The advancements in early diagnosis and the development of novel anti-cancer strategies have significantly enhanced breast cancer therapy, leading to improved five-year survival rates in the majority of patients with breast cancer. However, individuals diagnosed with triple-negative (TNBC) and HER2-positive breast cancers face lower survival rates due to the absence of effective therapeutic targets and prognostic markers (Hunter et al. 2020; Waks and Winer 2019). Therefore, the understanding of the pathogenesis of breast cancer is essential in order to identify potential targets for not only diagnosis and prognosis but also for the development of personalized treatment strategies.

Kinetochore-localized astrin/SPAG5-binding protein (KNSTRN), also known as C15orf23 or SKAP, is a mitosis-related protein that is not only a primary component of the mitotic spindle but also binds directly to microtubules (Friese et al. 2016), thereby largely contributing to cell division (Friese et al. 2016; Wong et al. 2019). The phosphorylation of KNSTRN via glycogen synthase kinase-3β (GSK3-β) in mitosis facilitates the interaction between KNSTRN and Kinesin Family Member 2B (Kif2b), thereby regulating chromosome segregation (Qin et al. 2016). Moreover, the astrin-SKAP complex localizes to the microtubule plus ends and facilitates chromosome alignment to regulate spindle position (Dunsch et al. 2011; Kern et al. 2016). Additionally, the astrin-SKAP complex binds directly to both microtubules and the nuclear division cycle 80 (Ndc80) complex to regulate mitosis (Kern et al. 2017). Furthermore, KNSTRN has been reported to promote metaphase-to-anaphase transition and chromosome segregation during mitosis (Fang et al. 2009). The role of KNSTRN in tumors involves the promotion of tumorigenesis and gemcitabine resistance through the activation of AKT (also known as protein kinase B) in bladder cancers (Xiong et al. 2021). Deng et al. utilized bioinformatics analyses to demonstrate that KNSTRN exhibited high expression levels in lung adenocarcinomas and was significantly associated with unfavorable prognosis (Lee et al. 2014). Furthermore, comprehensive whole-exome sequence analyses identified KNSTRN as one of the top three frequently mutated genes in cutaneous squamous cell carcinomas (SCCs) (Lee et al. 2014). However, the expression and biological roles of KNSTRN in breast cancers remain largely unknown.

The tumor microenvironment plays a key role in initiating and developing human malignancies (Hui and Chen 2015; Klemm and Joyce 2015; Zou et al. 2023). The study conducted by Deng et al., utilizing data from The Cancer Genome Atlas (TCGA), revealed a positive correlation between KNSTRN and Th2 cells as well as CD56dim natural killer (NK) cells in lung adenocarcinoma. Furthermore, it was observed that KNSTRN exhibited significant differential expression across various immune cell types (Deng et al. 2021). The findings suggest a crucial involvement of KNSTRN in immune infiltration, highlighting its pivotal role. Additionally, numerous studies have demonstrated the significance of immune infiltration in breast cancer proliferation, metastasis, and drug resistance (Mehraj et al. 2021; Deepak et al. 2020). However, to the best of our knowledge, no studies have yet found a relationship between KNSTRN and immune infiltration in breast cancer.

**Fig. 1:**
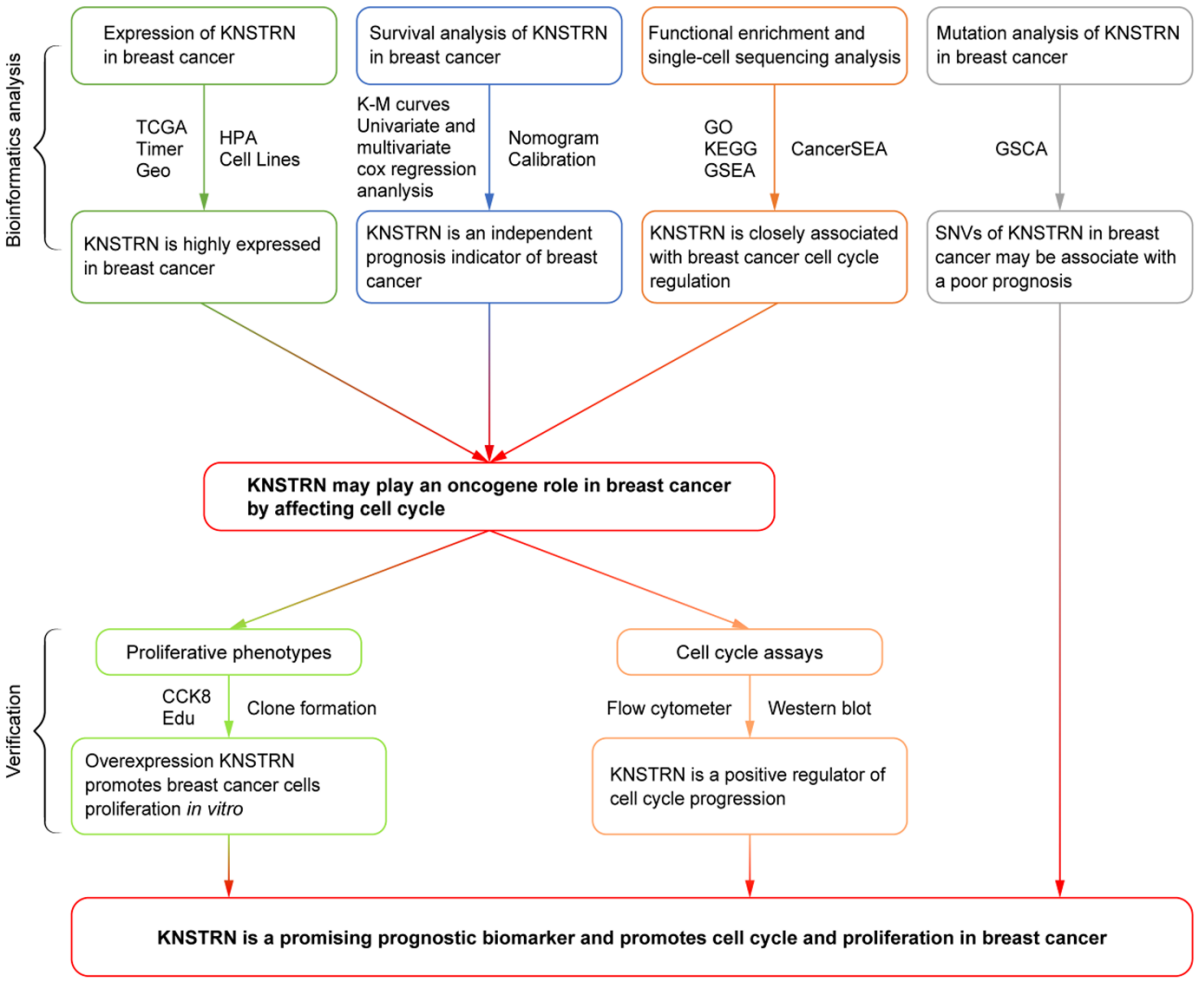
Analysis of the flowchart

In the present study, we conducted a comprehensive analysis of KNSTRN expression, prognostic implications, correlation with immune infiltration, expression-associated genes, and regulated signaling pathways to elucidate its role in cell cycle regulation using bioinformatics and in vitro functional experiments (Fig. 1). We aimed to investigate the diagnostic and prognostic values of KNSTRN in breast cancer patients, explore its correlation with immune infiltration, and demonstrate the effect of KNSTRN on the proliferation of breast cancer cells.

## Materials and Methods

### Data Acquisition and Processing

The Tumor IMmune Estimation Resource (TIMER) algorithm database was used to estimate KNSTRN gene expression levels in various types of cancers (http://www.linkedomics.org) (Li et al. 2020). The clinical information and high-throughput RNA sequencing data of breast cancer patients were obtained from two sources: The Cancer Genome Atlas (TCGA) database (https://cancergenome.nih.gov), which provided 1109 breast cancer samples, 113 adjacent non-tumor samples, and 112 paired breast cancer samples, and the Gene Expression Omnibus (GEO) (https://www.ncbi.nlm.nih.gov/geo/), where GSE45827 dataset was extracted, consisting of 130 breast cancer tissues, 14 breast cancer cell lines, and 11 normal breast tissues (Edgar et al. 2002; Clarke et al. 2013). The Human Protein Atlas (https://www.proteinatlas.org/) is an extensive bioinformatics web resource dedicated to systematically mapping all human proteins. Immunohistochemical images from the Human Protein Atlas were utilized to compare the protein expression levels of KNSTRN in normal and breast cancer tissues (Thul and Lindskog 2018). We also used DiseaseMeth version 3.0 (http://diseasemeth.edbc.org/) (Xing et al. 2022) to investigate the relationship between the expression of KNSTRN and its methylation level. Furthermore, we analyzed the correlation between KNSTRN and SPAG5 and Ki67 using cBioPortal (https://www.cbioportal.org/) (Cerami et al. 2012; Gao et al. 2013).

We obtained 20 breast cancer tissues and para-cancerous tissues from the biological sample bank of Qingyuan People’s Hospital for the purpose of detecting mRNA and protein levels of KNSTRN (15 cases for mRNA detection and 5 cases for protein detection). These human studies were conducted in strict accordance with the principles outlined in the Declaration of Helsinki and received approval from the Ethics Committee of Qingyuan People’s Hospital (IRB-2022-010).

We procured a human breast cancer tissue microarray, comprising 30 paraffin-embedded breast cancer tissues, from Shanghai Outdo Biotech Co, Ltd. (#HBreD030PG01, Shanghai, China) to assess the correlation between the protein expression levels of KNSTRN and Ki67 through immunohistochemistry. The KNSTRN antibody (NBP1-94007, diluted 1:200) and Ki67 antibody (ab15580, diluted 1:500) were employed as primary antibodies. The remaining experimental procedures were conducted following previously described methods (Lei et al. 2022).

### Survival Analysis

To investigate the correlation between KNSTRN expression and prognosis in breast cancer patients, we categorized the cancer samples into high- and low-expression groups based on the median KNSTRN expression. Subsequently, Kaplan–Meier plots (http://kmplot.com/analysis) were constructed to analyze the survival curves (Győrffy 2021). Univariate and multivariate Cox regression analyses were performed to evaluate the prognostic potential of KNSTRN in breast cancer (Liu et al. 2018). The forest plot was used to show each variable’s p-value, hazard ratio (HR), and 95% confidence interval using the ‘forest plot’ R package. A nomogram was constructed to predict the three-year and five-year overall recurrence. The nomogram provides a graphical representation of the factors used to calculate an individual patient’s risk of recurrence, based on the points associated with each risk factor using the ‛Rms and Survival’ R package. The calibration curve was assessed graphically by comparing the probabilities predicted by the nomogram with the observed ratios, and the 45° line represented the optimal predicted value.

### Single-Nucleotide Variant Analysis

Gene set cancer analysis (GSCA) (http://bioinfo.life.hust.edu.cn/gsca/#/) is an integrated platform for genomic, pharmacogenomic, and immunogenomic cancer analyses that provides comprehensive information on mRNA expression, mutations, immune infiltration, and drug resistance (Liu et al. 2018b). We used GSCA to analyze the frequency of single-nucleotide variants (SNVs) in KNSTRN in cancerous cells and its relationship with prognoses.

### Immune Infiltration Analysis

We utilized the “ESTIMATE” package to evaluate the association between KNSTRN expression and immune scores, stroma score, estimate scores, as well as tumor purity. Furthermore, their correlations were subsequently determined based on the Pearson correlation coefficient. We also analyzed the correlation between KNSTRN expression and different immune cell infiltration using ImmuCellAI (http://bioinfo.life.hust.edu.cn/ImmuCellAI#!/) (Miao et al. 2020, 2021).

### Differential Expression and Functional Enrichment Analysis

Breast cancers were divided into KNSTRN high- and low-expression clusters according to the median value normalized using the Z-score. The ‛DESeq2’ and ‛ggplot2’ R packages were utilized to identify differentially expressed genes (DEGs) and visualize their expression levels through a volcano plot (|log_2_FC|> 1.5, *p* < 0.05) (Love et al. 2014). The enrichment analyses of differentially expressed genes (DEGs) were conducted using the “clusterProfiler” package (Love et al. 2014) with default parameters, applying Gene Ontology (GO) and Kyoto Encyclopedia of Genes and Genomes (KEGG) databases. *p*-values were corrected through Benjamini–Hochberg correction, and results for *p*.adj < 0.1 were further visualized. The gene set enrichment analysis (GSEA) was conducted on all the differentially expressed genes (DEGs) using the ‛clusterProfiler’ package, with the c2.cp.v7.2.symbols.gm set as the reference gene set. The significance of enrichment was determined by a false discovery rate < 0.25 and p.adj < 0.05 (Subramanian et al. 2005).

### Single-Cell Sequencing Data Analysis

The CancerSEA database (http://biocc.hrbmu.edu.cn/CancerSEA/home.jsp) is the pioneering resource for single-cell sequencing, offering comprehensive insights into diverse functional states of cancer cells (Yuan et al. 2019). We retrieved data from CancerSEA that demonstrated the correlation between KNSTRN expression and various functional states of tumors, which were subsequently visualized on a heat map. Additionally, breast cancer single-cell sequencing data (Exp ID: EXP0052) was obtained from CancerSEA for the purpose of investigating the associations among KNSTRN expression, cell cycle progression, DNA damage response, proliferation rate, and DNA repair mechanisms in breast cancers using Spearman’s rank correlation test. The resulting t-SNE plots were directly acquired from the online CancerSEA platform.

### Cell Culture and Transfection

The cell lines, including MDA-MB-231, HS578T, UACC812, MCF7, T47D, HCC1954, and MCF10A, were obtained from the Shanghai Institute of Biological Sciences and the Chinese Academy of Sciences. MDA-MB-231, HS578T, UACC812, MCF7, and T47D cells were cultured in Dulbecco’s modified Eagle medium supplemented with 10% fetal bovine serum (Gibco). HCC1954 cells were cultured in RPMI1640 (Gibco) supplemented with 10% fetal bovine serum. The normal breast epithelial cell line MCF10A was cultured in mammary epithelial cell basal medium (Lonza). All cells were maintained at 37 °C under a 5% CO2 atmosphere. The siRNAs used in this study were purchased from GenePharma (Suzhou). According to the manufacturer’s instructions, Plasmid and siRNA transfection experiments were performed using Lipofectamine 3000 (Invitrogen). The KNSTRN gene was cloned into a TK-PCDH-copGFP-T2A-Puro vector between Nhel and Notl restriction sites. The siRNAs targeting KNSTRN had the following sequences: siRNA-KNSTRN#1: 5ʹ-GCUACAAACCACUGAGUAATT-3ʹ and siRNA-KNSTRN#2: 5ʹ-CCGAUUCCUAGAACAGCAATT-3ʹ.

### Cell Proliferation Assays

The effects of KNSTRN overexpression or knockdown on cell viability were measured using CCK-8 (Cell Counting Kit-8; Dojindo, Japan), clone formation assays, and 5-ethynyl-20-deoxyuridine (EdU) assays.

For CCK8 assays, 48 h after plasmid and siRNA transfection, transformants were inoculated into 96-well plates at a density of 2 × 10^3^ cells per well. At time points of 0, 24, 48, 72, and 96 h after inoculation, each well was supplemented with 10 µL of CCK8 reagent and incubated for a duration of two hours at a temperature of 37 °C. The absorbance value at the wavelength of 450 nm was subsequently measured (Tecan, Austria).

For clone formation assays, transformants were inoculated into six-well plates at a density of 2000 cells per well and cultured in Dulbecco’s modified Eagle medium supplemented with 20% fetal bovine serum for a duration of 2 weeks. Subsequently, the cells were washed twice with phosphate-buffered saline, fixed with 4% paraformaldehyde for a period of 20 min, and stained with 0.1% crystal violet solution for a duration of 5 min. The number of cell clones in each experimental group was quantified.

The rate of DNA synthesis in breast cancer cells (1 × 10^4^/well) was quantified using an EdU assay kit (Ribobio, China) following the manufacturer’s instructions for EdU assays. Proliferation activity was evaluated by determining the ratio of EdU-positive cells (red fluorescence) to Hoechst-stained cells (blue fluorescence). Experiments were conducted in triplicate.

### RNA Isolation and Real-Time Fluorescence Quantitative PCR

RNA from cells was extracted using a total isolation kit (Vazyme, China), according to the manufacturer’s instructions. RNA concentrations were measured using a Nanodrop One spectrophotometer (Thermo Fisher, USA). The RNA was subsequently reverse transcribed into cDNA using a cDNA synthesis kit (Vazyme). Real-time fluorescence quantitative PCR (qRT-PCR) was performed using an SYBR Green Master Mix Kit (Vazyme) with the primers: KNSTRN forward: 5ʹ-CCGCCTCGTTACGATGACC-3ʹ; KNSTRN reverse: 5ʹ-TGGCCCGAGTTTGTGTGTC-3ʹ and glyceraldehyde 3-phosphate dehydrogenase (GAPDH) forward: 5ʹ-GGTGTGAACCATGAGAAGTATGA-3ʹ; GAPDH reverse: 5ʹ-GAGTCCTTCCACGATACCAAAG-3ʹ. The expression level of the *GAPDH* gene was used for normalization. The real-time PCR amplification conditions were as follows: 95 °C for 30 s; 40 cycles of 95 °C for 10 s and 60 °C for 30 s, followed by collecting fluorescence; and finally melt curve at 95 °C for 15 s, 60 °C for 60 s, and 95 °C for 15 s. Experiments were performed in triplicate.

### Western Blotting

Cells (2 × 10^6^) were collected and lysed using radioimmunoprecipitation assay buffer (Absin, China) containing phenylmethylsulfonyl fluoride (Absin) and phosphatase inhibitor cocktails (Sigma, USA). Protein concentrations were determined using a BCA protein assay kit (Beyotime, China). For sodium dodecyl sulfate–polyacrylamide gel electrophoresis, the samples were supplemented with 3 × 10^4^ ng of protein and imprinted on polyvinylidene difluoride membranes (Millipore, Billerica, MA, USA). The membranes were then blocked with 5% skim milk for 1 h at room temperature and then incubated with the respective primary antibodies —KNSTRN (1:1000 dilution, #26189–1-AP, Proteintech, USA), cyclin A2 (1:1000 dilution, #4656, Cell Signaling Technology, USA), cyclin B1(1:1000 dilution, #12231, Cell Signaling Technology), cyclin D3 (1:1000 dilution, #2936, Cell Signaling Technology), cyclin E2 (1:1000 dilution, #4132, Cell Signaling Technology), CDK4 (1:1000 dilution, #12790, Cell Signaling Technology), CDK6 (1:1000 dilution, #3136, Cell Signaling Technology), p27^kip1^ (1:1000 dilution, #3686, Cell Signaling Technology), and β-actin (1:5000 dilution, #ab8226, Abcam, USA)—overnight at 4 °C. The membranes were then incubated with secondary antibodies conjugated to horseradish peroxidase (1:5000 dilution, #7076, #7074, Cell Signaling Technology) for 1 h at room temperature. Signals were detected using a chemiluminescence detection reagent (Millipore).

### Flow Cytometry for Cell Cycle Analysis

Forty-eight hour post-transfection with either plasmids or siRNA, the cells were harvested and fixed overnight at −20 °C in 75% ethanol. Following a phosphate-buffered saline wash, the cells were incubated with propidium iodide/RNase A solution (#abs50005, Absin, China) at 37 °C for 30 min, consistent with previous studies (Liu et al. 2021). Samples were analyzed within 5 h of staining using a flow cytometer (BD, USA), and data were analyzed using the FlowJo V10 software.

### Statistical Analysis

The experimental results were analyzed using the GraphPad Prism version 8.4.0 for Mac OS X (GraphPad Software, San Diego, CA, USA), using the average of three replicates. Data were tested for normal distribution (Kolmogorov–Smirnov test) and homogeneity of variance (Levene’s test). For the analysis of quantitative data from two independent samples, a two-tailed unpaired *t* test was used if the data met the normal distribution and the variances were equal, the Welch’s corrected unpaired *t* test was used if the data only met the normal distribution and the variances were not equal and a non-parametric test was used if the data did not meet the normal distribution. For two paired samples, the paired *t* test was used if the difference followed a normal distribution; if the difference did not follow a normal distribution, then the Wilcoxon rank sum test was used. Chi-square test or Fisher exact test was used for categorical variables. The Kaplan–Meier method was used to evaluate survival across groups, and the analysis of variance (ANOVA) was used to assess the significance of differences among various groups. Spearman’s rank correlation coefficient and Pearson correlation coefficient were used to assess the correlation between the two groups. The diagnostic accuracy was assessed by receiver operating characteristic (ROC) curve analysis. Results were considered significant at *P < 0.05, **P < 0.01, and ***P < 0.001.

## Results

### KNSTRN Is Significantly Highly Expressed in Breast Cancers and Serves as a Potential Pathological Biomarker for Diagnosis

**Fig. 2:**
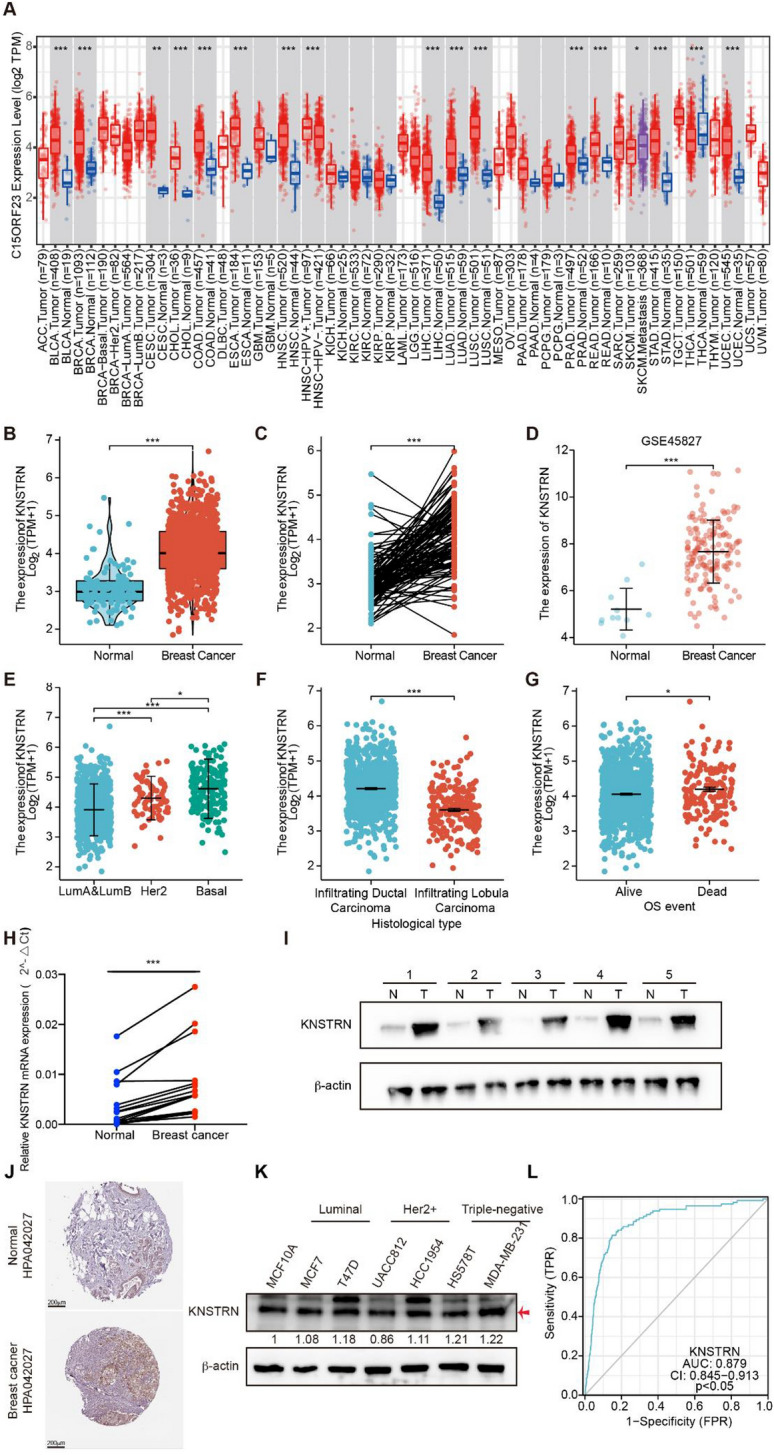
KNSTRN was significantly overexpressed in breast cancer tissues and cells. **(A)** The mRNA expression levels of KNSTRN in pan-cancer by TIMER database. **(B, C)** The mRNA expression levels of KNSTRN in normal breast tissues and non-paired **(B)** or paired **(C)** breast cancer tissues. **(D)** The mRNA expression of KNSTRN in unpaired normal tissues and breast cancer tissues from the Gene Expression Omnibus (GEO) database (No. GSE42587). **(C-G)** The mRNA expression of KNSTRN in breast cancer patients with different clinical characteristics in The Cancer Genome Atlas (TCGA) [molecular subtypes **(E)**, histological type **(F)**, OS event**(G)**]. **(H)** The mRNA level of KNSTRN was upregulated in 15 breast cancer tissue samples by RT-qPCR. **(I)** The protein level of KNSTRN was upregulated in 5 breast cancer tissue samples by Western blot. **(J)** Validation of KNSTRN expression levels in breast cancer tissues using the Human Protein Atlas database. **(K)** Western blot detecting the protein expression of KNSTRN in MCF10A and different breast cancer cell lines. **(L)** Diagnostic value of KNSTRN in breast cancer patients by ROC curve analysis. *p < 0.05, **p < 0.01, and ***p < 0.001. MCF10A, normal breast epithelial cell line. ROC, receiver operating characteristic

The process of this study was visually represented through the construction of a flowchart. (Fig. 1). The TIMER database was utilized to investigate the mRNA expression of KNSTRN in various cancer types. In comparison to normal tissues, elevated levels of KNSTRN expression were observed in bladder urothelial carcinoma, breast invasive carcinoma, cervical squamous cell carcinoma and endocervical adenocarcinoma, cholangiocarcinoma, colon adenocarcinoma, esophageal carcinoma, head and neck squamous cell carcinoma, liver hepatocellular carcinoma, lung adenocarcinomas, lung squamous cell carcinoma, prostate adenocarcinomas, rectal adenocarcinoma, stomach adenocarcinoma, and uterine corpus endometrial carcinoma. Conversely, decreased expression was noted in thyroid carcinomas (Fig. 2A). These results were corroborated by the transcriptome data of patients with breast cancer from TCGA, which revealed that KNSTRN mRNA levels were significantly higher in breast cancer than in normal tissues, regardless of whether they were non-paired or paired primary tumors (*p* < 0.001, Fig. 2B, C). The results from the analysis of the GEO dataset (No. GSE42587) further validated the higher mRNA expression of KNSTRN in the breast cancer tissues (*p* < 0.001, Fig. 2D).

To investigate the correlation between KNSTRN expression and clinical characteristics in breast cancer patients, we conducted an analysis of KNSTRN mRNA expression levels across different TCGA clinical categories (Table 1). The highest and lowest expression of KNSTRN were in TNBC and luminal type breast cancers, respectively (*p* < 0.05, Fig. 2E), and was significantly elevated in both infiltrating ductal carcinomas (*p* < 0.001, Fig. 2F) and patients who died (*p* < 0.027, Fig. 2G), suggesting that high expression of KNSTRN contributed to the malignant transformation of breast cancers.

**Table 1:** Correlation between KNSTRN expression and clinical features in breast cancer patients

Characteristic	Low expression of KNSTRN	High expression of KNSTRN	p
n	541	542	
T stage, n (%)			** < 0.001**
T1	174 (16.1%)	103 (9.5%)	
T2	279 (25.8%)	350 (32.4%)	
T3	74 (6.9%)	65 (6%)	
T4	14 (1.3%)	21 (1.9%)	
N stage, n (%)			0.089
N0	261 (24.5%)	253 (23.8%)	
N1	182 (17.1%)	176 (16.5%)	
N2	46 (4.3%)	70 (6.6%)	
N3	43 (4%)	33 (3.1%)	
M stage, n (%)			0.595
M0	438 (47.5%)	464 (50.3%)	
M1	8 (0.9%)	12 (1.3%)	
Pathologic stage, n (%)			**0.003**
Stage I	113 (10.7%)	68 (6.4%)	
Stage II	291 (27.5%)	328 (30.9%)	
Stage III	120 (11.3%)	122 (11.5%)	
Stage IV	8 (0.8%)	10 (0.9%)	
Age, n (%)			** < 0.001**
< = 60	271 (25%)	330 (30.5%)	
> 60	270 (24.9%)	212 (19.6%)	
Histological type, n (%)			** < 0.001**
Infiltrating Ductal Carcinoma	323 (33.1%)	449 (46%)	
Infiltrating Lobular Carcinoma	158 (16.2%)	47 (4.8%)	
PR status, n (%)			** < 0.001**
Negative	118 (11.4%)	224 (21.7%)	
Indeterminate	2 (0.2%)	2 (0.2%)	
Positive	397 (38.4%)	291 (28.1%)	
ER status, n (%)			** < 0.001**
Negative	64 (6.2%)	176 (17%)	
Indeterminate	0 (0%)	2 (0.2%)	
Positive	453 (43.8%)	340 (32.9%)	
HER2 status, n (%)			0.446
Negative	285 (39.2%)	273 (37.6%)	
Indeterminate	4 (0.6%)	8 (1.1%)	
Positive	77 (10.6%)	80 (11%)	
PAM50, n (%)			** < 0.001**
Normal	30 (2.8%)	10 (0.9%)	
LumA	407 (37.6%)	155 (14.3%)	
LumB	39 (3.6%)	165 (15.2%)	
Her2	26 (2.4%)	56 (5.2%)	
Basal	39 (3.6%)	156 (14.4%)	
Age, median (IQR)	60 (50, 68)	56 (48, 66)	**0.001**

Twenty pairs of paired tumor and adjacent human tissue samples were used to detect the mRNA and protein expression levels of KNSTRN in patients with breast cancer. The findings revealed a significant increase in both mRNA and protein expression levels of KNSTRN among breast cancer patients compared to their adjacent tissues (Fig. 2H, I). The immunohistochemical results obtained from the Human Protein Atlas also confirmed this conclusion (Fig. 2J). The expression of KNSTRN in breast cancer cell lines was assessed by western blotting. The levels of KNSTRN expression were found to be higher in MCF7, T47D, HCC1954, HS578T, and MDA-MB-231 cells compared to the normal breast epithelial cell line MCF10A, with the exception of UACC812 cells (Fig. 2K). These results support the hypothesis that KNSTRN is highly expressed at both the mRNA and protein levels in breast cancers. To further investigate the mechanisms by which KNSTRN expression is abnormally upregulated in breast cancer tissues, we examined the relationship between KNSTRN methylation and expression levels using DiseaseMeth, version 3.0. KNSTRN methylation levels did not differ significantly between tumor and para-carcinoma tissues (Supplementary Table S1), suggesting that the abnormally high expression of KNSTRN in breast cancer is independent of its methylation levels. Furthermore, according to ROC curve analysis, KNSTRN is a potential diagnostic biomarker for breast cancer (AUC = 0.879, Fig. 2L). The collective findings suggest that KNSTRN exhibits high expression levels across various cancer types, including breast cancer, thereby indicating its potential as a pathological biomarker for the accurate diagnosis of breast cancer.

### KNSTRN Is an Independent Prognosis Indicator of Breast Cancer

**Fig. 3:**
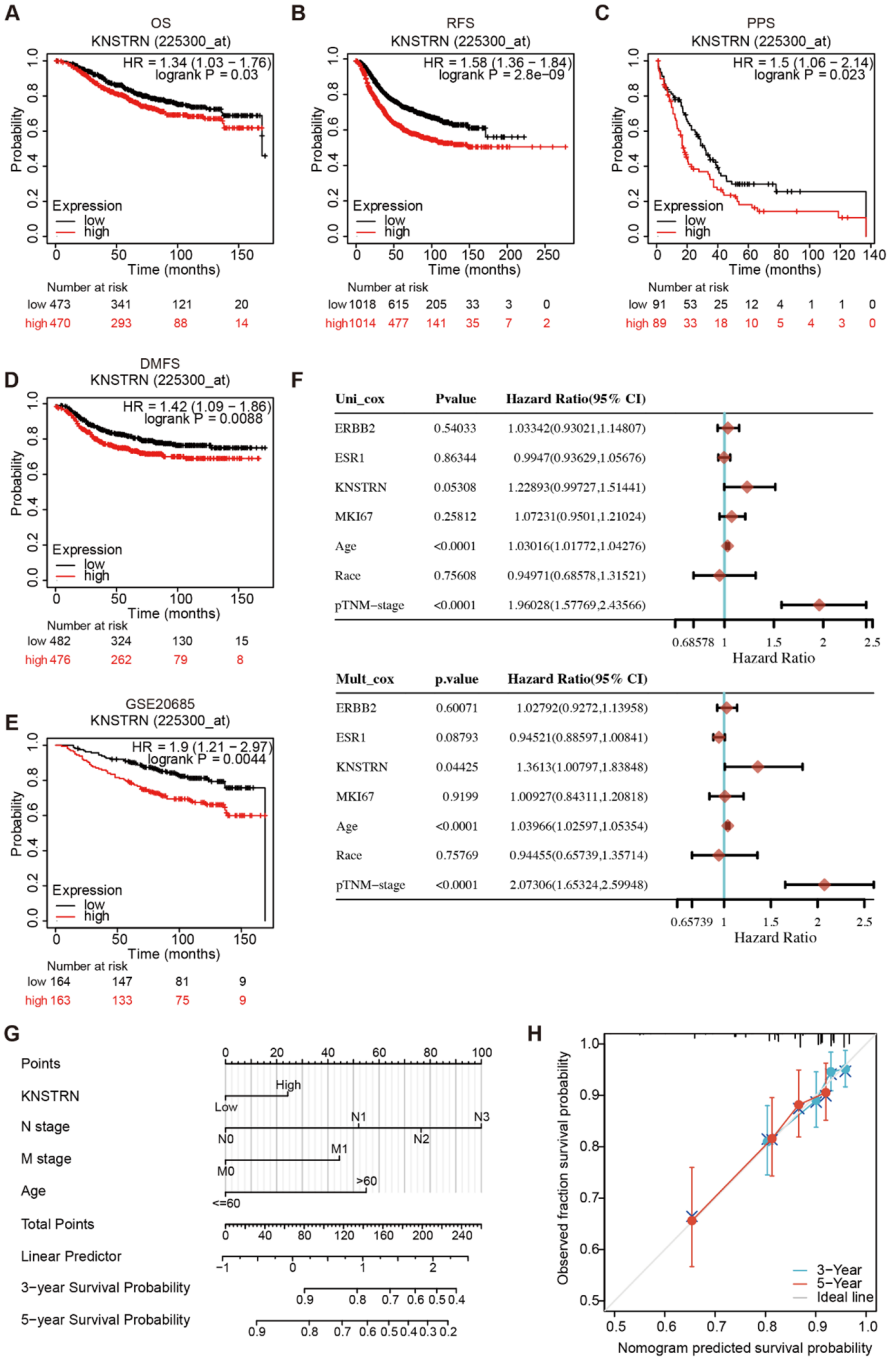
High expression of KNSTRN was significantly associated with poor prognosis and was an independent prognosis factor in breast cancer. **(A-D)** Correlation between KNSTRN expression and prognosis by Kaplan–Meier plotter database [overall survival (OS) **(A)**, relapse-free survival (RFS) **(B)**, post-progression survival (PPS) **(C)**, and distant metastases-free survival (DMFS) **(D)**]. **(E)** Correlation between KNSTRN expression and OS in GSE20685 dataset. **(F)** The forest map results of the univariate and multivariate survival analyses of OS among breast cancer patients are shown. **(G)** Nomogram predicting 3- and 5-year overall survival of breast cancer patients.** (H)** The calibration chart shows the predictive performance of the nomogram model

To investigate the association between KNSTRN expression and the prognosis of patients with breast cancer, we performed a survival analysis using the Kaplan–Meier method. Our findings revealed a significant and inverse correlation between elevated KNSTRN expression and overall survival (OS), relapse-free survival, post-progression survival, as well as distant metastasis-free survival in breast cancer patients (Fig. 3A–D). The analysis of data from the GEO dataset (No. GSE20685) confirmed that the OS of patients with high KNSTRN expression was significantly shorter than that of patients with low KNSTRN expression (Fig. 3E). We then analyzed the relationship between KNSTRN expression and the outcome of patients with breast cancer in different clinical subgroups. The results showed that high KNSTRN expression was significantly associated with a worse outcome for patients with breast cancer in T2 (HR = 1.62, *p* = 0.031), N1 (HR = 1.74, *p* = 0.04), N3 (HR = 5.96, *p* = 0.004), M0 (HR = 1.51, *p* = 0.023), Stage II (HR = 1.64, *p* = 0.04), Stage III (HR = 2.70, *p* = 0.016), and those over 60 years of age (HR = 1.85, *p* = 0.008) (Table 2). KNSTRN is a SPAG5-binding protein, and SPAG5 has been validated as an independent prognostic biomarker in breast cancer (Abdel-Fatah et al. 2016; He et al. 2020). Through cBioPortal, we found a significant positive correlation between KNSTRN and SPAG5 (Supplementary Fig. 1A) and between KNSTRN and Ki67 (Supplementary Fig. 1B), which was further demonstrated by immunohistochemistry (*R* = 0.612, Supplementary Fig. 1C). The above findings suggest that KNSTRN exhibits a significant prognostic value and holds great potential as a prognostic biomarker in breast cancer.

**Table 2:** The relationship between KNSTRN expression and overall survival (OS) in different clinical subgroups of breast cancer patients

Characteristic	N (%)	HR (95% Cl)	p
T stage			
T1	277 (25.6%)	0.90(0.44–1.82)	0.761
T2	629 (58.2%)	1.62(1.04–2.52)	**0.031**
T3	139 (12,9%)	2.64(0.99–7.06)	0.053
T4	35 (3.2%)	0.80(0.25–2.52)	0.698
N stage			
N0	514 (48.3%)	1.72(0.95–3.11)	0.074
N1	358 (33.6%)	1.74(1.03–2.94)	**0.04**
N2	116 (10.9%)	1.58(0.65–3.85)	0.311
N3	76 (7.1%)	5.96(1.79–19.78)	**0.004**
M stage			
M0	902 (97.8%)	1.51(1.06–2.15)	**0.023**
M1	20 (2.2%)	0.52(0.14–1.88)	0.316
Pathologic stage			
Stage I	181 (17.1%)	1.50(0.55–4.07)	0.423
Stage II	619 (58.4%)	1.64(1.02–2.63)	**0.04**
Stage III	242 (22.8%)	2.70(1.20–6.10)	**0.016**
Stage IV	18 (1.7%)	2.32(0.68–7.92)	0.178
Age			
< = 60	601 (55.5%)	1.55(0.92–2.63)	0.101
> 60	482 (44.5%)	1.85(1.18–2.90)	**0.008**

Subsequently, we performed univariate and multivariate Cox regression analyses on the relationships among KNSTRN, HER2 (ERBB2), ER (ESR1), and Ki67 (MKI67) expression, clinical factors (age, race, and pTNM-stage), and OS in patients with breast cancer. Univariate Cox analysis showed that age (*p* < 0.0001) and pTNM-stage (*p* < 0.0001) were significantly correlated with OS in breast cancers, whereas KNSTRN expression was almost significantly correlated with OS in breast cancers (*p* = 0.05308). Interestingly, multivariate Cox regression analysis revealed a significant association between KNSTRN expression and overall survival (*p* = 0.04425), suggesting that KNSTRN expression may be an independent prognostic factor for breast cancer (Fig. 3F). We also constructed a nomogram table to assess the three- and five-year OS probability of patients with breast cancer (Fig. 3G). The calibration chart shows that the nomogram model has high prediction accuracy (Fig. 3H). Our findings indicate that elevated expression of KNSTRN is significantly associated with an unfavorable prognosis and serves as an independent prognostic factor in patients diagnosed with breast cancer.

### Relationship Between KNSTRN Expression and Immune Infiltration

**Fig. 4:**
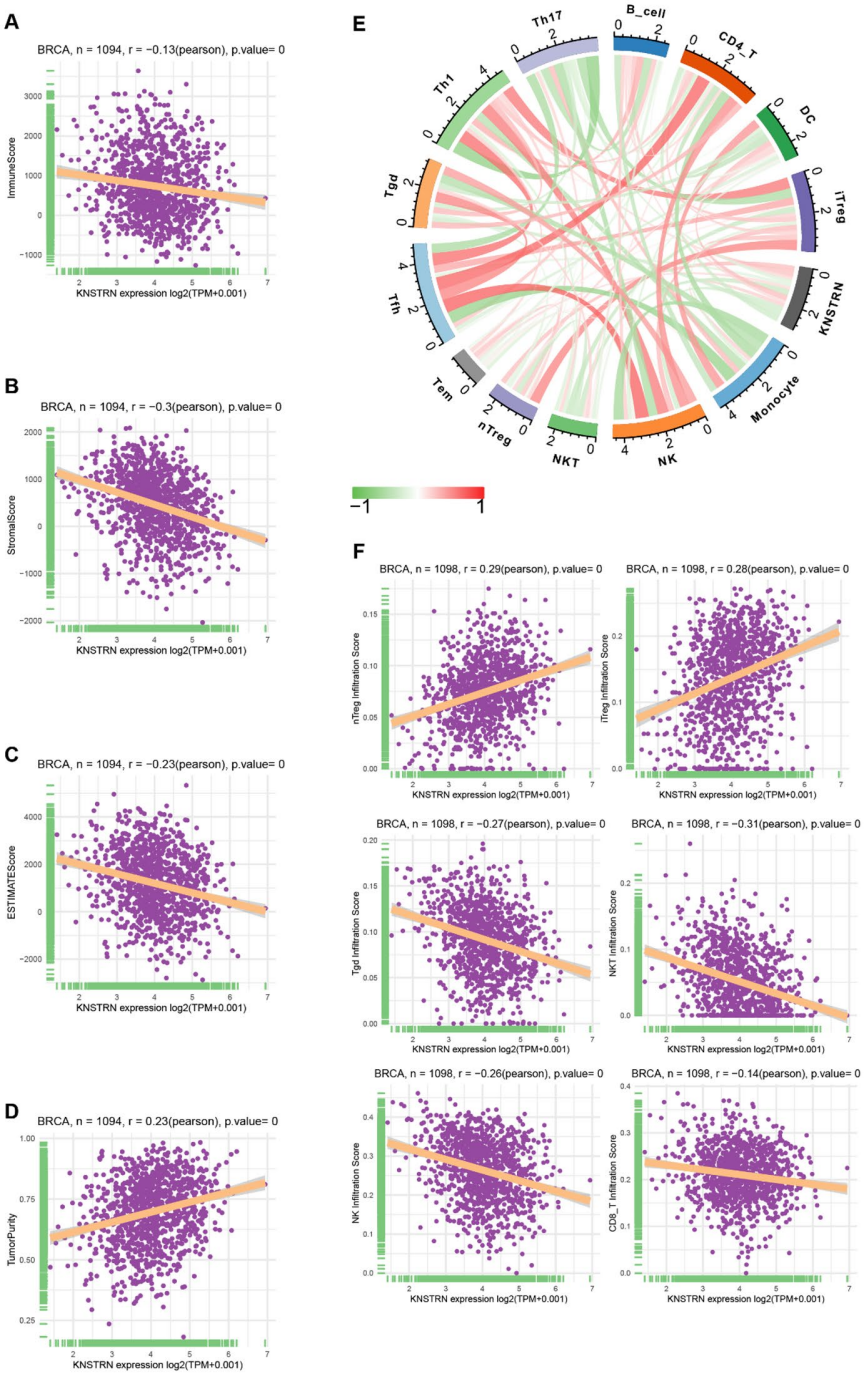
Correlation between KNSTRN expression and immune infiltration. **(A-D)** The relationship between KNSTRN expression and immune score **(A)**, stroma score **(B)**, estimate score **(C)**, and tumor purity **(D)**. **(E)** The relevancy between KNSTRN expression and different immune cells. **(F)** Relevance of KNSTRN expression to nTreg infiltration, iTreg infiltration, Tgd infiltration, NKT infiltration, NK infiltration, and CD8-T infiltration

The infiltration of immune cells plays a crucial role in the pathogenesis of breast cancer (Mehraj et al. 2021; Deepak et al. 2020). Therefore, we investigated the correlation between KNSTRN expression and immune infiltration in breast cancer across multiple GEO datasets. As depicted in Supplementary Fig. 2, KNSTRN expression was higher in Tprolif cells while showing lower levels in regulatory (Treg) and NK cells. Subsequently, we examined the relationship between KNSTRN expression and immune scores, stromal scores, and tumor purity using the “ESTIMATE” package. Notably, elevated KNSTRN expression was found to be inversely correlated with immune scores and stromal scores, suggesting a reduction of immune and stromal cells within the tumor microenvironment when KNSTRN is highly expressed (Fig. 4A–D). The correlation between KNSTRN and different immune cells is shown in Fig. 4E. KNSTRN expression was significantly and positively correlated with natural regulatory T-cell (nTreg) and induced regulatory T-cell (iTreg) infiltration score, while its expression was significantly and negatively correlated with Tγδ (Tgd), NKT, NK, and CD8-T infiltration score in breast cancer (Fig. 4F). These results suggest that KNSTRN may be associated with increased Treg infiltration, an immune cell that promotes tumor development and decreased Tgd, NK infiltration, a tumor-killing cell in breast cancer.

### SNVs of KNSTRN in Breast Cancer Might Be Associated with a Poor Prognosis

**Fig. 5:**
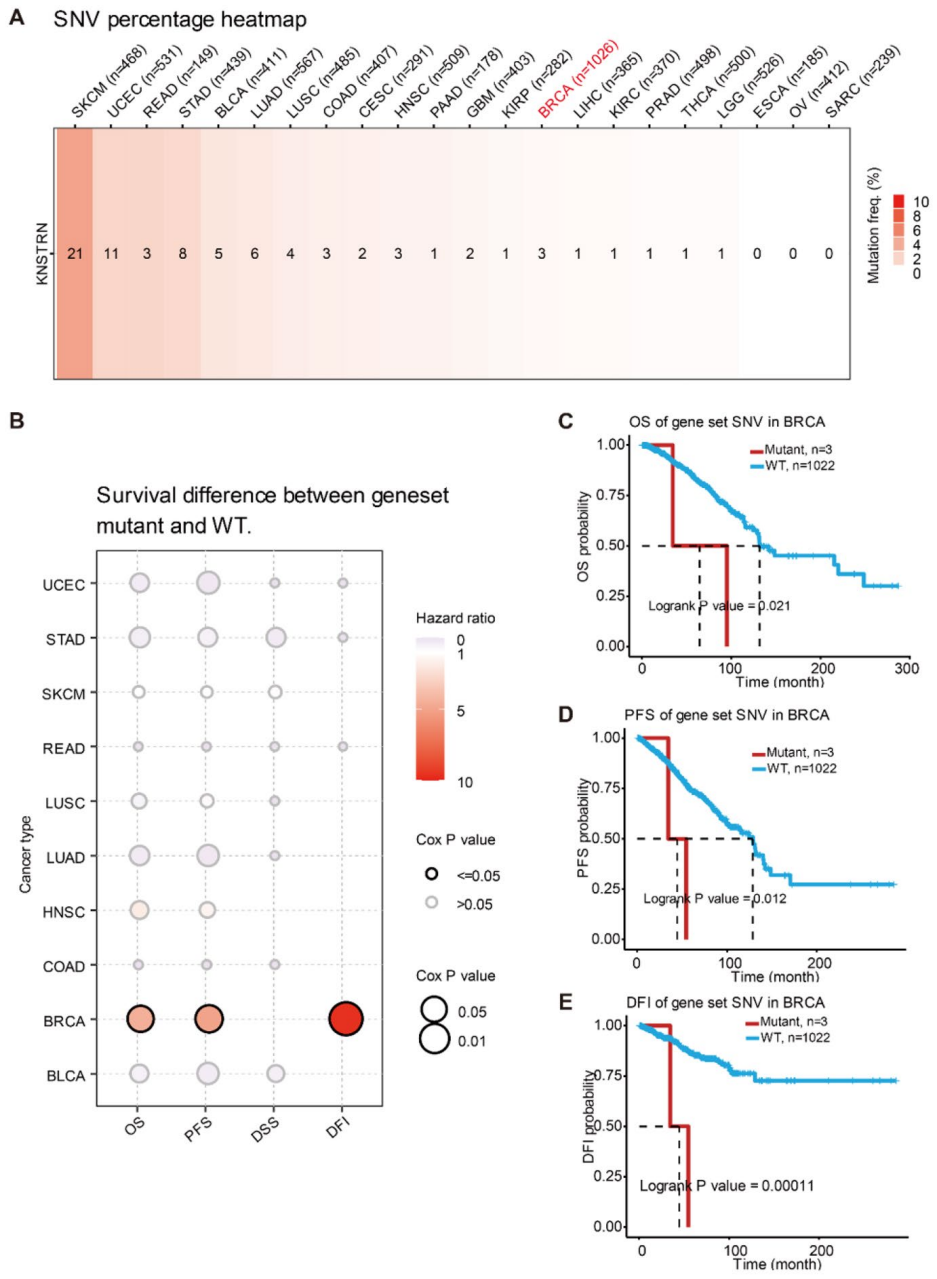
SNVs of KNSTRN were associated with a poor prognosis in breast cancer. **(A)** The frequency of SNV in *KNSTRN* in pan-cancer. **(B)** Relationship between the SNV of KNSTRN and prognosis in a variety of tumors. **(C-E)** Correlation between the SNV of KNSTRN and prognosis in breast cancer [overall survival (OS) **(C),** progression-free survival (PFS) **(D)**, and disease-free interval (DFI) **(E)**]. SNV, single-nucleotide variation

Gene mutations contribute to the initiation, progression, and diagnosis of tumors. Hence, we initially examined the prevalence of single-nucleotide variants (SNVs) in KNSTRN across pan-cancer samples. The highest incidence of SNVs of KNSTRN was 21% in skin cutaneous melanoma, while breast cancer exhibited a comparatively lower frequency of only 3% (Fig. 5A). Subsequently, we analyzed the relationship between SNVs and survival. KNSTRN mutations were significantly associated with poor prognosis in patients with breast cancer (Fig. 5B). Survival curves suggested that SNVs of KNSTRN in breast cancer were significantly and negatively correlated with OS, progression-free survival, and disease-free interval (Fig. 5C–E). The findings suggest a potential association between SNVs of KNSTRN and an unfavorable prognosis in breast cancer. However, due to the limited sample size of patients harboring this mutation, further validation through large-scale sequencing is warranted.

### Differential Expressed Genes Associated with KNSTRN and Their Functional Enrichment Analysis in Breast Cancer

**Fig. 6:**
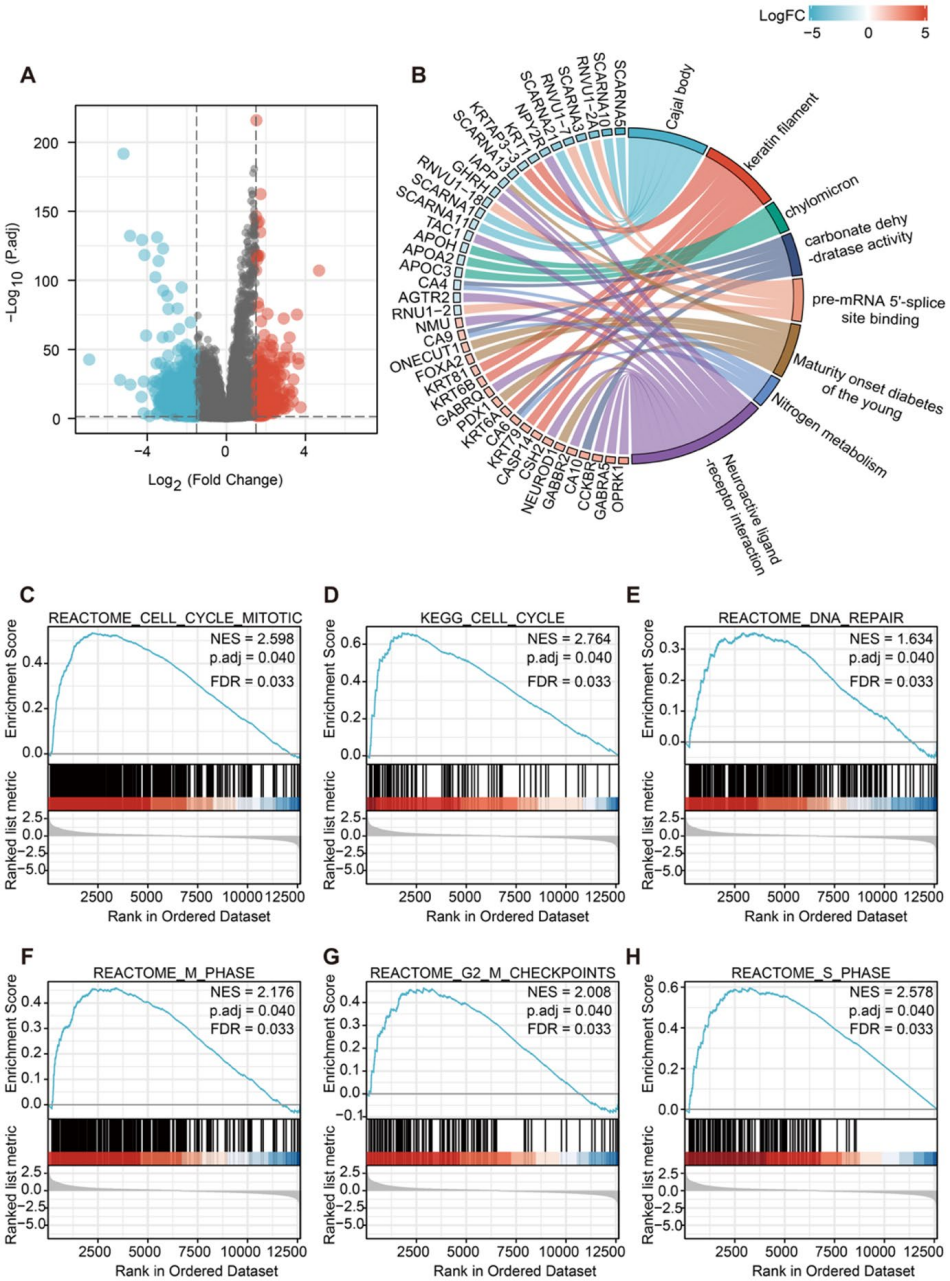
Differentially expressed genes (DEGs) related to KNSTRN expression and functional enrichment analysis. **(A)** The volcanic plot of DEGs, red dots indicate significantly upregulated genes, blue dots indicate significantly downregulated genes. **(B)** GO and KEGG enrichment analysis of significant DEGs (|log2FC|> 1.5, *p*. adj < 0.05) and the *p*-values were corrected by Benjamini—Hochberg correction, and results for *p*.adj < 0.1 were further visualized. **(C-H)** Functional pathway analysis of all DEGs by GSEA. GSEA revealed that the enriched gene sets were significantly associated with **(C)** cell cycle mitotic, **(D)** cell cycle, **(E)** DNA repair, **(F)** M phase, **(G)** G2-M checkpoints, and **(H)** S phase, false discovery rate (FDR) < 0.25 and *p*. adj < 0.05 were assumed to be a significant enrichment. GO, Gene Ontology. KEGG, Kyoto Encyclopedia of Genes and Genomes. GSEA, Gene Set Enrichment Analysis. NES, normalized enrichment score. FDR, false discovery rate

Based on the findings of the mRNA, protein, prognostic, and immune infiltration analyses of KNSTRN, our hypothesis is that KNSTRN plays an oncogenic role in breast cancer. However, the precise molecular mechanism underlying the impact of KNSTRN on tumorigenesis remains elusive. Therefore, we analyzed DEGs associated with KNSTRN using TCGA. Using |log_2_FC|> 1.5 and *p.adj* < 0.05 as the screening criteria, we identified 1682 DEGs, including 352 upregulated and 1330 downregulated genes (Fig. 6A). The GO and KEGG analyses of the DEGs using the R ‘clusterProfiler’ package revealed that the significant DEGs were mostly enriched in signaling pathways, specifically nitrogen metabolism and neuroactive ligand–receptor interactions (Fig. 6B). Through GSEA, we found that the DEGs were mainly enriched in the cell cycle, DNA repair, M phase, G2-M checkpoints, and S phase processes (Fig. 6C–H). These findings imply that KNSTRN plays a pivotal role in governing the progression of the cell cycle in breast cancer.

### KNSTRN Is Strongly Associated with Cell Cycle in Breast Cancer

**Fig. 7:**
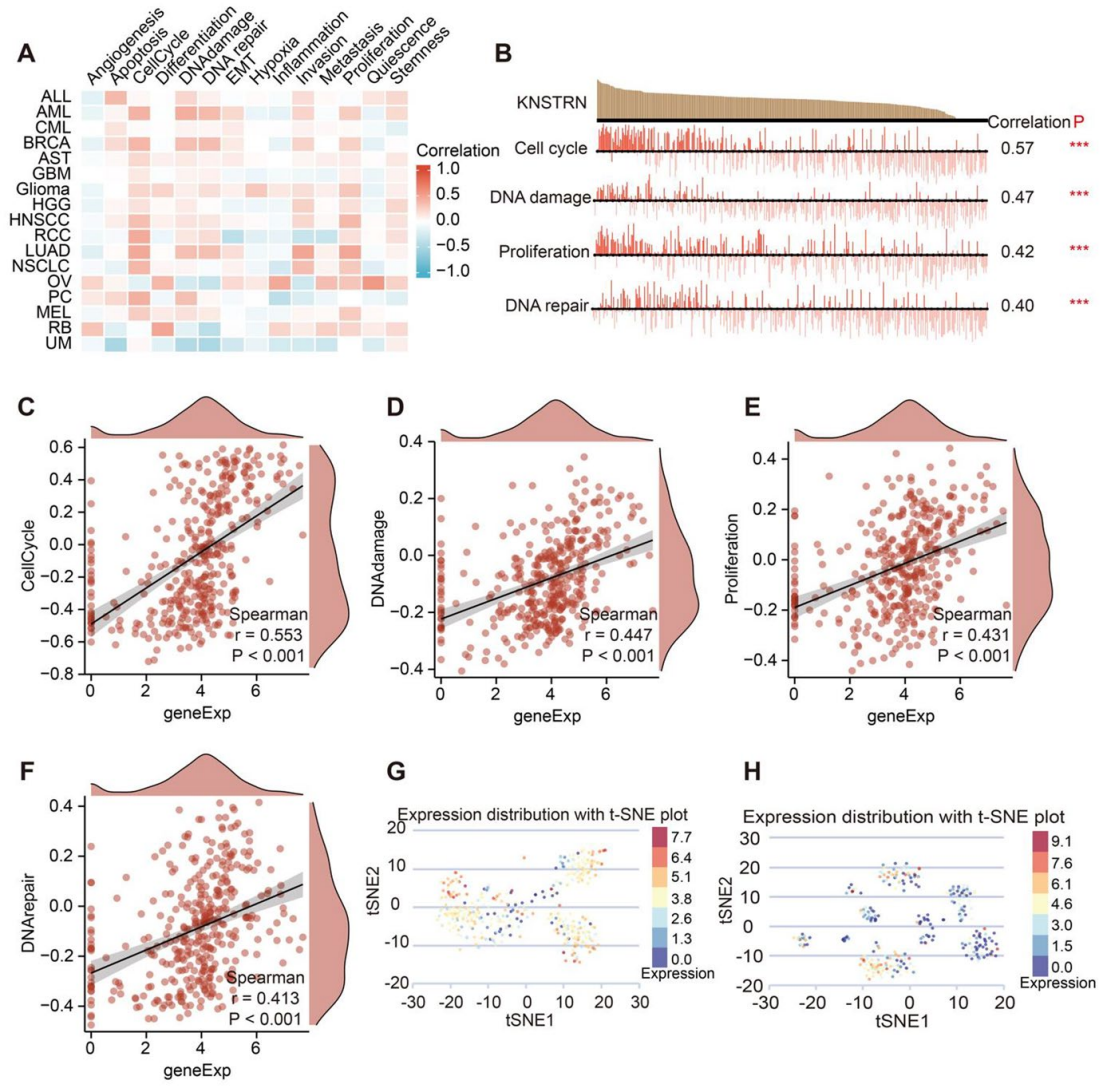
The expression of KNSTRN in single-cell sequencing and its correlation with the functional status of breast cancer. **(A)** Heatmap showing the correlation between KNSTRN and different tumor statuses based on CancerSEA database. **(B)** Correlation between KNSTRN expression and four significantly functional statuses in breast cancer. **(C, D)** Correlation of KNSTRN expression with the cell cycle **(C)**, DNA damage **(D)**, Proliferation **(E)**, and DNA repair **(F)** by Spearman’s rank correlation test. **(G, H)** T-SNE diagram demonstrated KNSTRN expression profiles in single cells of breast cancer based on the CancerSEA database [Cell group ID: EXP0052 **(G)**, EXP0053**(H)**]. ***p < 0.001

The composition of breast tumors encompasses various cellular components, including neoplastic cells, vascular elements, immune cell populations, and fibroblasts (Bahcecioglu et al. 2020). The utilization of single-cell sequencing enables a more comprehensive comprehension of the multitude of cellular states, heterogeneity within cell populations, and the underlying mechanisms driving oncogenesis in breast cancer (Ding et al. 2020; Li et al. 2022). Therefore, we analyzed KNSTRN expression at the single-cell level in different cancers and explored the relationship between KNSTRN expression and tumor functional status using the Cancer SEA database. KNSTRN was correlated with the cell cycle in various cancers, including acute myeloid leukemia (Cor = 0.403,* p* = 0.007), lung adenocarcinoma (Cor = 0.45, *p* < 0.001), non-small cell lung cancer (Cor = 0.37, *p* < 0.001), renal cell carcinoma (Cor = 0.44, *p* = 0.001), and breast cancer (Cor = 0.38, *p* < 0.001) (Fig. 7A). KNSTRN was negatively correlated with DNA repair (Cor =  − 0.401, *p* < 0.001) in retinoblastoma and apoptosis (Cor =  − 0.50, *p* < 0.001) in uveal melanoma (Fig. 7A). In the single-cell transcriptomic profile of breast cancer, KNSTRN was significantly and positively correlated with the cell cycle, DNA damage, proliferation, and DNA repair at the single-cell level (Fig. 7B–F). The expression profile of KNSTRN in single cells of breast cancer suggested that KNSTRN was highly expressed in breast cancer cells at the single-cell level (Fig. 7G, H).

As KNSTRN is closely related to the cell cycle, we analyzed the relationship between KNSTRN expression and the key cell cycle markers on TCGA. The expression of KNSTRN showed a strong and positive correlation with cell cycle markers, including the G1 phase regulators CDK4 and CDK6; S phase regulators CDK2 and CCNA2; G1/S transition regulators CCNE1 and CCNE2; and G2/M transition regulators CDK1 (Supplementary Fig. 3). These findings suggest that KNSTRN plays a crucial role, either directly or indirectly, in various cell cycle phases and serves as an indispensable regulator of breast cancer cell cycle progression.

### KNSTRN Promotes Breast Cancer Cell Proliferation by Regulating the Cell Cycle

**Fig. 8:**
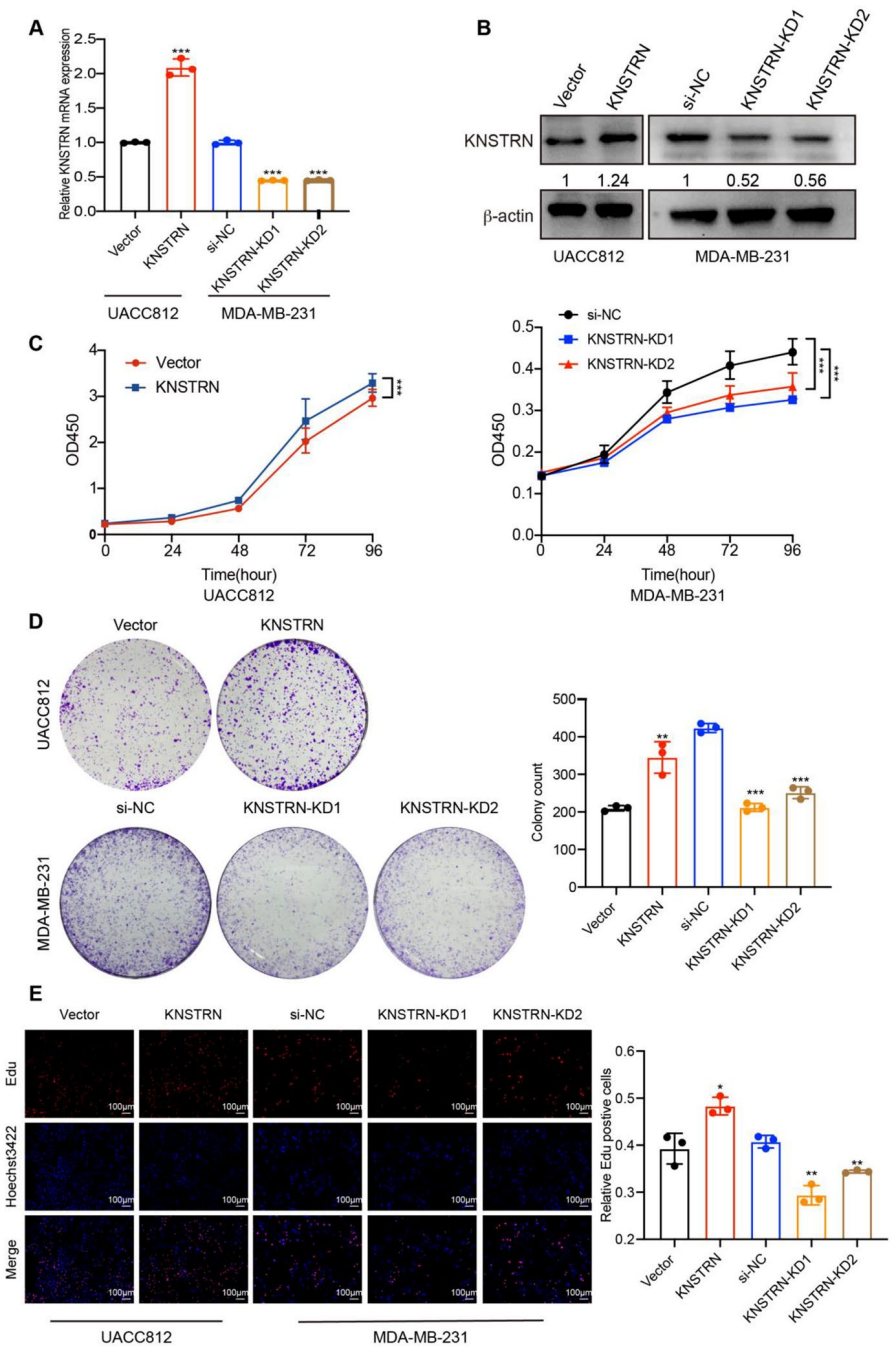
KNSTRN promoted breast cancer cell proliferation in vitro*. ***(A, B)** The efficiency of KNSTRN overexpression in UACC812 and KNSTRN knockdown in MDA-MB-231 at mRNA and protein levels were validated by RT-qPCR **(A)** and western blot **(B**). **(C)** CCK-8 assays measuring the cell proliferation kinetics after overexpression in UACC812(left) and knockdown in MDA-MB-231(right). **(D)** Colony formation assays evaluated the proliferative capacity after KNSTRN overexpression in UACC812 (up) and KNSTRN knockdown in MDA-MB-231 (down). **(E)** Edu assays detecting the DNA synthesized rate after KNSTRN overexpression in UACC812 (left) and KNSTRN knockdown in MDA-MB-231(right). *p < 0.05, **p < 0.01, and ***p < 0.001. CCK-8, Cell Counting Kit 8. Edu, 5-ethynyl-20-deoxyuridine

To validate the effect of KNSTRN on the cell cycle and cell proliferation in breast cancer cells, we measured the proliferative phenotypes and the expression of cell cycle regulators in breast cancer cells. The expression level of KNSTRN was the lowest in UACC812 cells and the highest in MDA-MB-231 cells, as indicated by Fig. 2K. Therefore, we chose UACC812 for KNSTRN overexpression and MDA-MB-231 for KNSTRN knockdown. KNSTRN overexpression in UACC812 and silencing in MDA-MB-231 were confirmed through qRT-PCR and western blotting (Fig. 8A, B). The proliferation of UACC812 cells was enhanced by KNSTRN overexpression, as evidenced by consistent results from CCK8, EdU, and colony formation assays. Conversely, siRNA-mediated knockdown of KNSTRN suppressed the proliferation of MDA-MB-231 cells (Fig. 8C–E).

**Fig. 9:**
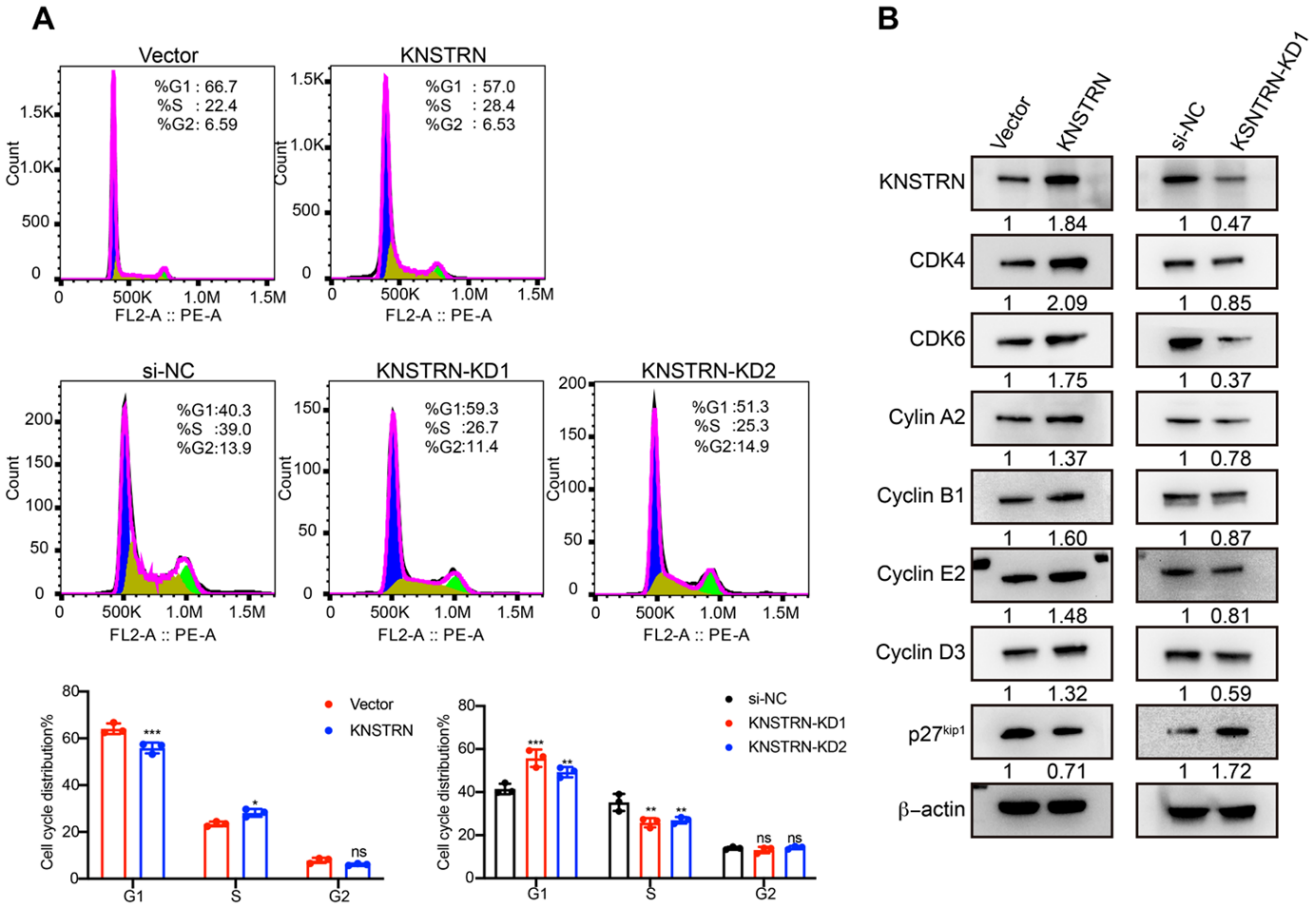
KNSTRN promoted the G1/S transition and cell cycle progression in breast cancer. **(A)** The proportion of KNSTRN overexpressing cells and KNSTRN knockdown cells in the G1, S, and G2-M phases of the cell cycle was analyzed by flow cytometry. **(B)** The relative expression levels of CDK4, CDK6, cyclin A2, cyclin B1, cyclin E2, cyclin D3, and p27^kip1^ were examined by western blot after KNSTRN overexpression in MCF7 and KNSTRN silencing in MDA-MB-231. *p < 0.05, **p < 0.01, and ***p < 0.001

We then investigated the role of KNSTRN in cell cycle progression and its underlying molecular mechanisms. Flow cytometry assays revealed that KNSTRN overexpression decreased the proportion of UACC812 cells in the G1 phase, and the proportion of MDA-MB-231 cells in the G1 phase was increased significantly when KNSTRN was silenced. Conversely, KNSTRN overexpression increased the proportion of UACC812 cells, whereas KNSTRN silencing reduced the proportion of MDA-MB-231 cells in S phase. However, the proportion of UACC812 and MDA-MB-231 cells in the G2 phase was unaffected by KNSTRN silencing or overexpression (Fig. 9A). Western blot analysis revealed that KNSTRN overexpression elevated the expression levels of the G1 phase regulators CDK4, CDK6, and cyclin D3; G1/S transition regulator cyclin E2; S phase regulator cyclinA2; and M phase regulator cyclin B1 but suppressed the expression of p27^kip1^, which blocked the cyclin-CDK binding. Conversely, KNSTRN silencing produced opposite results to those of KNSTRN overexpression (Fig. 9B).

Collectively, these findings indicate that KNSTRN serves as a positive regulator of cell cycle progression. Inhibition of KNSTRN expression not only leads to cell cycle arrest at the G1-S transition but also diminishes the proliferative capacity of breast cancer cells.

## Discussion

KNSTRN encodes a protein that regulates mitotic chromosome segregation (Lee et al. 2016). Few studies have shown that KNSTRN plays an important role in tumors (Xiong et al. 2021; Deng et al. 2021; Lee et al. 2016; Jaju et al. 2015; Knstrn Deemed an Oncogene 2014), but the present study is the first to reveal the functions of KNSTRN in breast cancer.

We conducted an analysis of KNSTRN expression levels in various types of cancers using the TIMER database and observed a significant upregulation of KNSTRN expression in several tumors, including bladder urothelial carcinoma, breast cancer, and cholangiocarcinoma. However, we also noted a downregulation of KNSTRN expression in thyroid carcinoma, suggesting potential variations in the functional roles of KNSTRN across different tumor types. Notably, triple-negative breast cancer (TNBC) exhibits heightened aggressiveness and metastatic potential compared to other subtypes of breast cancer (Garrido-Castro et al. 2019). We observed higher expression of KNSTRN mRNA and protein in triple-negative breast cancer (TNBC) compared to luminal and HER2 types, indicating a potential role of elevated KNSTRN expression in driving the malignant transformation of breast cancers. The receiver operating characteristic (ROC) analysis demonstrated an area under the curve (AUC) value of 0.879 for diagnosing breast cancer, suggesting that KNSTRN holds promise as a potential diagnostic biomarker. Additionally, our findings revealed a significant association between high KNSTRN expression and unfavorable overall survival, relapse-free survival, post-progression survival, and distant metastasis-free survival in patients with breast cancer. Furthermore, multivariate Cox regression analysis confirmed that high KNSTRN expression independently served as a prognostic factor. Therefore, KNSTRN represents a promising biomarker for both diagnosis and prognosis assessments in breast cancer.

KNSTRN is an essential component of the mitotic spindle that affects the cell cycle by contributing to chromosome alignment, accurate chromosome segregation, and maintenance of the spindle pole structure (Dunsch et al. 2011; Fang et al. 2009; Huang et al. 2012; Wang et al. 2012). In bladder cancer, KNSTRN affects the cell cycle by regulating the expression of cyclin D1 and CDK2 (Xiong et al. 2021). The present study demonstrated the association of KNSTRN with the cell cycle through GSEA and single-cell analyses. Additionally, flow cytometry revealed that KNSTRN significantly accelerated the G1/S transition. These findings strongly suggest a pivotal role for KNSTRN in regulating the cell cycle in breast cancer, thereby necessitating further investigations into its involvement across diverse tumor types.

As important components of cell cycle mechanisms, cyclins and their associated cyclin-dependent kinases regulate the mammalian cell cycle and promote cell cycle progression (Malumbres and Barbacid 2009; Sherr et al. 2016). Using western blotting, we demonstrated that KNSTRN overexpression elevated the expression of positive cell cycle regulators, such as CDK4, CDK6, and cyclin D3, and KNSTRN silencing produced contrasting results. Cyclin D-CDK4/6 drives the G1 to S phase transition by phosphorylating and inactivating retinoblastoma protein (Gao et al. 2020). Cylin E2 is not only stable in the S phase but is also independent of transcription and degradation, thus contributing to aberrant proliferation and genomic instability in breast cancers (Lee et al. 2020). During the S phase, cyclin A2 is restricted to the nucleus, and during the S/G2 phase transition, a portion of cyclin A2 is transferred to the cytoplasm to phosphorylate Aurora Borealis (Bora), which activates polo-like kinase 1 (PLK1) and promotes the cell cycle (Silva Cascales et al. 2021). Cylin B1 is a key regulator of G2/M transition, and Cyclin B1 overexpression accelerates mitosis and promotes excessive cell proliferation (Lv et al. 2020). By increasing the expression of these proteins, KNSTRN is evidently involved in different phases of the cell cycle and, thus, promotes the proliferation of breast cancer cells. Furthermore, we found that KNSTRN negatively regulated p27^Kip1^ expression. P27^Kip1^ is a cyclin-dependent kinase inhibitor that acts extensively on CDK-cyclin complexes, thereby inhibiting their activity (Bencivenga et al. 2017; Besson et al. 2008). As KNSTRN activates AKT, which phosphorylates p27^Kip1^ and inhibits its anti-proliferative effect, and the expression of KNSTRN is positively correlated with AKT serine/threonine kinase 1 (AKT1) (Xiong et al. 2021; Huo et al. 2019; Fresno Vara et al. 2004; Hinz and Jücker 2019; Min et al. 2004), we speculate that the inhibitory effect on p27^Kip1^ expression is caused by KNSTRN-mediated activation of AKT. Further experiments are required to verify this hypothesis.

Immune infiltration in breast cancer has been reported to have an impact on the proliferation and metastasis as well as the prognosis of patients with breast cancer (Wang et al. 2021; Burugu et al. 2017; Liu et al. 2022; Ye et al. 2023). In the present study, we found that KNSTRN expression was significantly positively correlated with Treg infiltration and negatively correlated with the infiltration of tumor-killing cells, such as Tgd and NK cells. Tregs can suppress the activity of effector T cells and other immune cells, which are important mediators of peripheral tolerance and prevent adverse immune responses (Mittal et al. 2018). Enhanced function and increased number of infiltrating Tregs in the tumor immune microenvironment limit the anti-tumor immune response and promote tumor angiogenesis and growth (Sharabi et al. 2018). Overexpression of the glycoprotein-A complex-based (GARP)/TGF-β axis could promote breast cancer progression through the expansion of Treg cells in the tumor microenvironment (Metelli et al. 2016). Upregulation of Treg was also observed in samples from 72 patients with early-stage breast cancer and was associated with tumor progression (Kim et al. 2013). Therefore, we speculate that besides accelerating the cell cycle, KNSTRN may also promote breast cancer progression by enhancing the infiltration of Treg cells.

Tregs are regarded as a prospective target for breast cancer treatment. However, selective and specifically targeted approaches for Treg depletion are lacking. Since KNSTRN expression is strongly positively correlated with Treg infiltration in breast cancer, targeting KNSTRN may be an effective strategy for depleting Tregs in breast cancer.

A large-scale pan-cancer study suggested that SNVs are a major driver of mutations in most cancers (Macintyre et al. 2016; Ciriello et al. 2013). SNVs in KNSTRN enhance tumorigenesis in SCCs. Lee et al. reported that multiple mutations of KNSTRN occurred in 19% of SCCs and a C → T transition that created the Ser24Phe mutation was most relevant to cancer because it disrupted chromatid cohesion in normal cells and enhanced tumorigenesis (Lee et al. 2014). Similarly, a PCR single-strand conformation polymorphism analysis, using SCCs tissues from 2229 Korean patients, showed that the KNSTRN Ser24Phe mutation was found specifically in SCCs (Lee et al. 2016). Schmitz et al. reported that a C → A transition that caused the Ala40Glu mutation in KNSTRN in SCCs, which was less frequent than the Ser24Phe mutation, was associated with the invasiveness of SCCs (Schmitz et al. 2019). In the present study, we found that the frequency of SNVs in KNSTRN was only 3% in breast cancers. However, the SNVs in KNSTRN were significantly positively associated with poor prognosis in patients with breast cancer, suggesting that these SNVs lead to an increase in transcriptional intensity and protein expression of KNSTRN, thus exacerbating the malignant transformation of breast cancer cells. The limited availability of data on KNSTRN mutations in breast cancer patients necessitates further investigation into the detection of KNSTRN alleles in large clinical samples, identification of mutation types and loci, and elucidation of variable splicing patterns in KNSTRN transcripts.

This study has several limitations. Firstly, the diagnostic efficacy of KNSTRN was not validated through pathological diagnosis of large-scale breast cancer samples. Secondly, the in vivo effects of KNSTRN on breast cancer cell cycle and cell proliferation were not investigated. We aim to conduct more comprehensive research in this area in future studies.

## Conclusion

Ultimately, our findings demonstrate that KNSTRN is a potential biomarker for the diagnosis and prognosis of breast cancer due to immune infiltration and proliferation within the context of breast cancer. Moreover, KNSTRN functions as an oncogene specific to breast cancer, whereby its heightened expression expedites G1/S transition and fosters cellular proliferation. Consequently, KNSTRN exhibits promising prospects as a diagnostic and prognostic marker for monitoring patients with breast cancer.

## Supplementary Information

Below is the link to the electronic supplementary material.

Supplementary file1 (DOCX 15680 KB)

## Data Availability

The datasets provided for this study can be found and accessed in online databases. These online databases were accessible from the following addresses. Tumor Immune Estimation Recourse (TIMER) database: http://www.linkedomics.org; The Cancer Genome Atlas (TCGA): https://cancergenome.nih.gov; Gene Expression Omnibus (GEO): https://www.ncbi.nlm.nih.gov/geo/; Human Protein Atlas (HPA): https://www.proteinatlas.org/; DiseaseMeth version 3.0: http://diseasemeth.edbc.org/; cBioPortal database: https://www.cbioportal.org/; Kaplan–Meier plotter: http://kmplot.com/analysis; Gene Set Cancer Analysis (GSCA): http://bioinfo.life.hust.edu.cn/GSCA/#/; ImmuCellAI: http://bioinfo.life.hust.edu.cn/ImmuCellAI#!/; TISIDB: http://cis.hku.hk/TISIDB/index.php; and CancerSEA: http://biocc.hrbmu.edu.cn/CancerSEA/.

## Abbreviations

GEO: Gene Expression Omnibus
GO: Gene Ontology
GSCA: Gene set cancer analysis
GSEA: Gene set enrichment analysis
HR: Hazard ratio
KEGG: Kyoto Encyclopedia of Genes and Genomes
NK: Natural killer
OS: Overall survival
ROC: Receiver operating characteristic
SCCs: Squamous cell carcinomas
SNVs: Single-nucleotide variants
TCGA: The Cancer Genome Atlas
TIMER: Tumor Immune Estimation Recourse
TNBC: Triple-negative breast cancer
