# Supplementary material for: KNSTRN Is a Prognostic Biomarker That Is Correlated with Immune Infiltration in Breast Cancer and Promotes Cell Cycle and Proliferation
Source: Biochem Genet. 2024 Jan 10;62(5):3709–39. doi: 10.1007/s10528-023-10615-2 (PMC11427568; doi:10.1007/s10528-023-10615-2)
Supplement: Supplementary file 1 — Supplementary file1 (DOCX 15680 KB) [file 10528_2023_10615_MOESM1_ESM.docx]

Supplementary Information

# Supplementary Figures and Tables

## Supplementary Figures


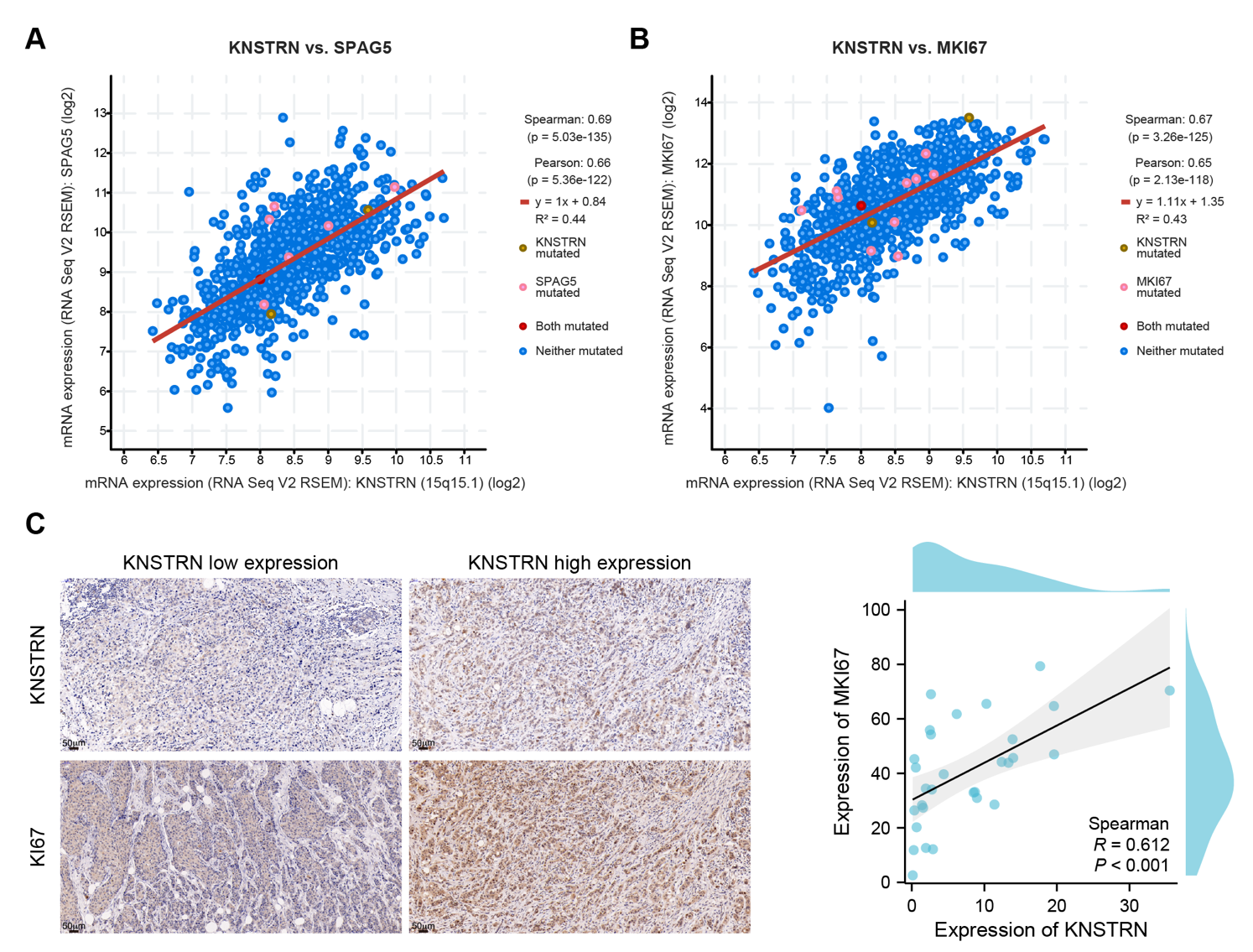


**Supplementary Figure 1.** The correlation between KNSTRN and SPAG5 (**A**) and Ki67 (**B**) through the cBioPortal database (<https://www.cbioportal.org/>). **(C)** Immunohistochemical analysis of the protein expression levels of KNSTRN and Ki67 and correlation between KNSTRN and Ki67 using the Spearman's rank correlation test.

**
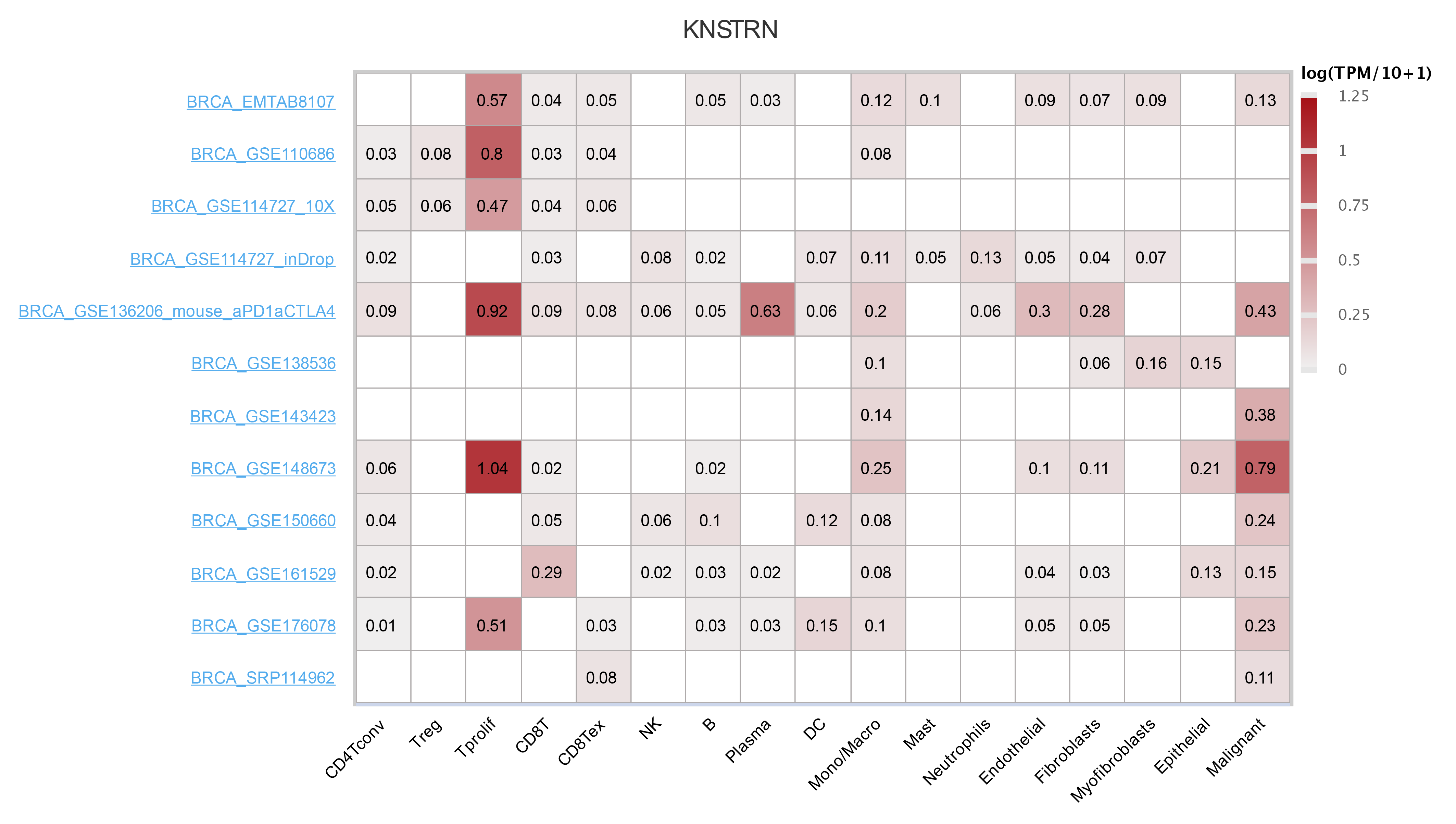
**

**Supplementary Figure 2.** Expression of KNSTRN in different immune cells.


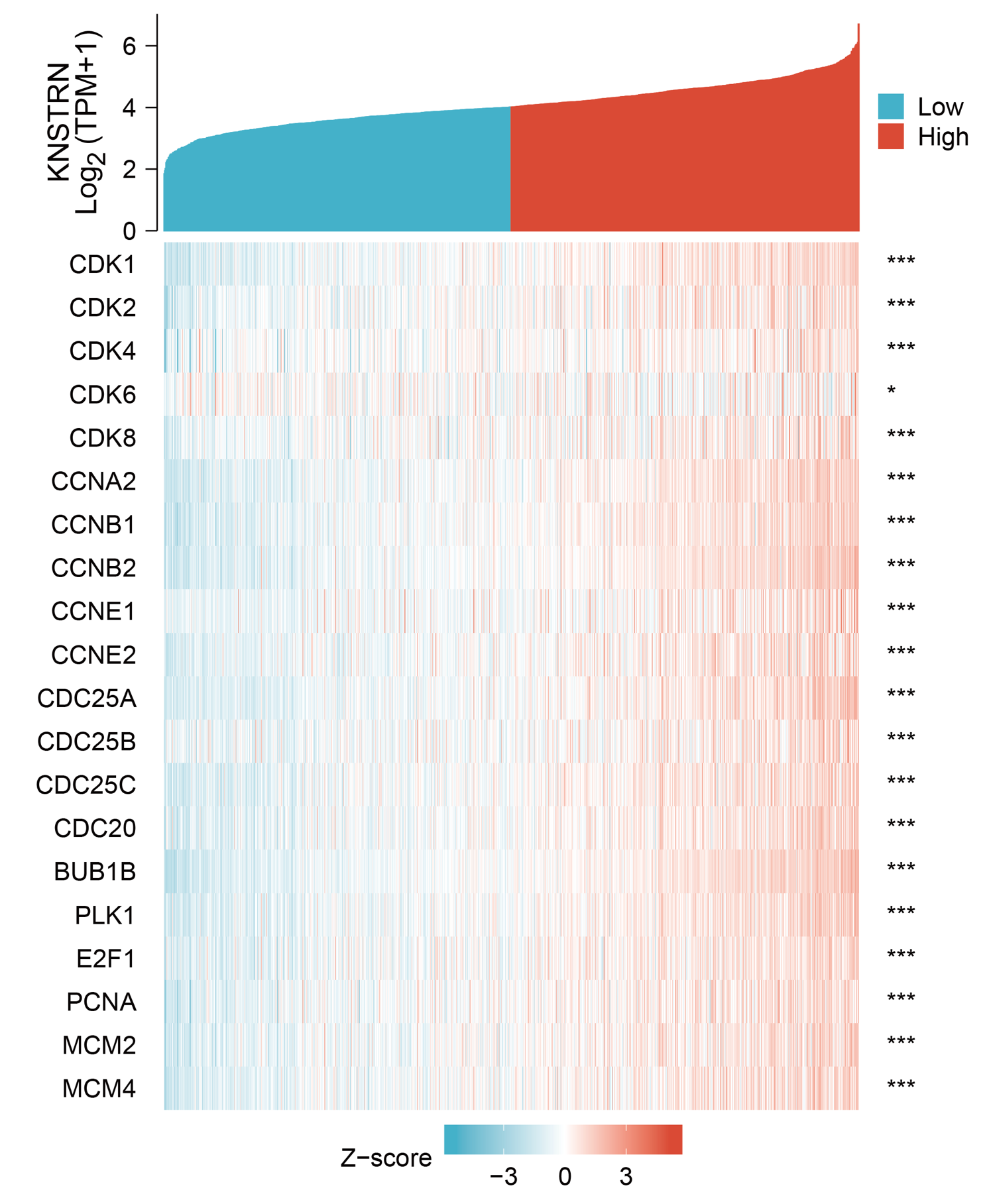


**Supplementary Figure 3.** Correlation between KNSTRN expression and key cell cycle biomarkers by TCGA data in breast cancer. **p*<0.05, ***p*<0.01, ****p*<0.001.

## Supplementary Tables

**Supplementary Table 1. Methylation analysis of KNSTRN in Breast invasion carcinoma using DiseaseMeth version**

| diseaseName | genomic region | transcript | gene | p-value | meanMethylDisea-meanMethylNormal |
| --- | --- | --- | --- | --- | --- |
| Breast invasive carcinoma | chr15:40672921-40675421 | NM_001142761 | KNSTRN | 0.0517 | -0.043 |
| Breast invasive carcinoma | chr15:40672921-40675421 | NM_001142762 | KNSTRN | 0.0517 | -0.043 |
| Breast invasive carcinoma | chr15:40672921-40675421 | NM_033286 | KNSTRN | 0.0517 | -0.043 |
